# Boronic Acids as Prospective Inhibitors of Metallo-β-Lactamases: Efficient Chemical Reaction in the Enzymatic Active Site Revealed by Molecular Modeling

**DOI:** 10.3390/molecules26072026

**Published:** 2021-04-02

**Authors:** Alexandra V. Krivitskaya, Maria G. Khrenova

**Affiliations:** 1 Bach Institute of Biochemistry, Federal Research Centre “Fundamentals of Biotechnology” of the Russian Academy of Sciences, 119071 Moscow, Russia; al_krivickaya@mail.ru; 2 Department of Chemistry, Lomonosov Moscow State University, 119991 Moscow, Russia

**Keywords:** NDM-1, metallo-β-lactamase, bacterial resistance, benzo[b] thiophene, boronic acid inhibitor, QM/MM, QTAIM, QM/MM molecular dynamics

## Abstract

Boronic acids are prospective compounds in inhibition of metallo-β-lactamases as they form covalent adducts with the catalytic hydroxide anion in the enzymatic active site upon binding. We compare this chemical reaction in the active site of the New Delhi metallo-β-lactamase (NDM-1) with the hydrolysis of the antibacterial drug imipenem. The nucleophilic attack occurs with the energy barrier of 14 kcal/mol for imipenem and simultaneously upon binding a boronic acid inhibitor. A boron atom of an inhibitor exhibits stronger electrophilic properties than the carbonyl carbon atom of imipenem in a solution that is quantified by atomic Fukui indices. Upon forming the prereaction complex between NDM-1 and inhibitor, the lone electron pair of the nucleophile interacts with the vacant *p*-orbital of boron that facilitates the chemical reaction. We analyze a set of boronic acid compounds with the benzo[b]thiophene core complexed with the NDM-1 and propose quantitative structure-sroperty relationship (QSPR) equations that can predict IC50 values from the calculated descriptors of electron density. These relations are applied to classify other boronic acids with the same core found in the database of chemical compounds, PubChem, and proposed ourselves. We demonstrate that the IC50 values for all considered benzo[b]thiophene-containing boronic acid inhibitors are 30–70 μM.

## 1. Introduction

Bacterial resistance is known from the very beginning of the penicillin era [1,2,3,4]. One of the mechanisms is attributed to the hydrolytic activity of the bacterial enzymes towards β-lactams. These enzymes are called β-lactamases (BLs), and they hydrolyze the C–N bond of the β-lactam ring of antibiotics forming the inactivated compounds, β-amino acids [5,6]. The antibiotic hydrolysis is initiated by various nucleophiles depending on the particular type of β-lactamases [6,7]. The B class BLs are metallo-β-lactamases (MBLs) that comprise one or two zinc cations in the active site [7]. These are divided into three subclasses, B1, B2, and B3 [8,9]. The most clinically relevant representative in the B1 subclass is the NDM-1 (New Delhi metallo-β-lactamase-1) [10]. It demonstrates substrate promiscuity [11], including hydrolysis of carbapenems [12]. In addition, it causes particular alertness due to its high spread between different bacterial species by lateral gene transfer favored by globalization and travel [13].

**Figure 1:**
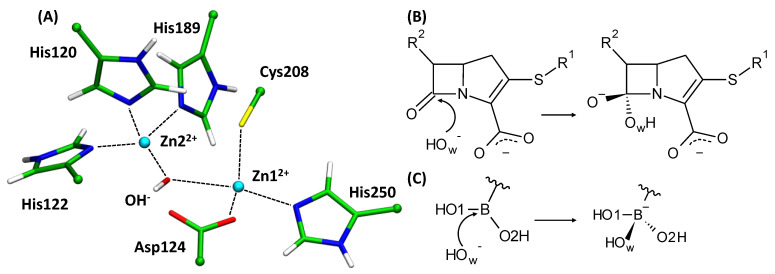
(**A**) The active site of NDM-1 metallo-β-lactamase (left). Nucleophilic attack step during (**B**) the hydrolysis of imipenem and (**C**) conversion of boronic acid to hydroxyboronate anion. The C_B_ atoms of the side chains of amino acids on the QM/MM border are shown in the ball’s representation. Color code: zinc—cyan, oxygen–red, sulfur—yellow, nitrogen—blue, hydrogen—white; carbon is green in complexes with imipenem and lavender in complexes with cpd5.

The active site of NDM-1 contains two zinc cations, Zn1^2+^ and Zn2^2+^, bridged by a catalytic hydroxide ion, OH^−^. The first zinc cation (Zn1^2+^) is coordinated by His120, His122 and His189, while the second zinc cation (Zn2^2+^) is coordinated by Asp124, Cys208 and His250 (Figure 1A). In the binding site, Zn1^2+^ orients and polarizes the carbonyl group of the substrate, facilitating the chemical reaction. The above-mentioned hydroxide anion is a nucleophile that initiates a chemical reaction by the attack on the β-lactam ring, eventually leading to substrate hydrolysis (Figure 1B) [8]. Since the discovery of NDM-1 [14], many inhibitors of various nature have been proposed. However, none of them has been clinically approved [8,15]. These are natural plant-based compounds [16,17], synthetic low molecular weight inhibitors [10,18,19,20,21,22], β-lactams [23,24,25,26], amino acid derivatives and peptides [27]. Boron-based inhibitors attract special attention [28,29,30]. Recent advances in developing the therapeutically relevant boron-containing compounds [31,32] even resulted in the FDA-approved anticancer drug bortezomib [33]. From the chemical viewpoint, these compounds are Lewis acids and can react reversibly with biologically relevant nucleophiles via their vacant *p*-orbitals [34,35,36,37]. They form covalent tetrahedral products, hydroxyboronate anions, upon reaction with the nucleophilic hydroxide anion in the active site of NDM-1 (Figure 1C) [18,35].

In this work, we focus on two features of boron-containing inhibitors. First, we compare interactions between the nucleophilic OH^−^ of the NDM-1 active site and its substrate imipenem with one of the boronic acid inhibitors (cpd5, according to Ref. [26]). To do this, we calculate the Gibbs energy profiles of the nucleophilic attack step using molecular dynamics (MD) simulations with the combined quantum mechanics/molecular mechanics QM(PBE0-D3/6-31G**)/MM potentials. Additionally, we compare the dynamic behavior of the corresponding reactant complexes with the same compounds in water solution, measuring atomic Fukui electrophilicity indices of the carbonyl carbon (in an imipenem) and boron (in a boronic acid inhibitor) atoms. Next, we consider five boronic acid inhibitors with the benzo[b]thiophene core with known IC50 values (concentration of inhibitor that results in 50% inhibition of the enzyme activity) [26] corresponding to the NDM-1 inhibition. We study covalent adducts, hydroxyboronate anions in the active site of NDM-1 by analysis of equilibrium geometry configurations of the NDM-1 with these compounds and suggest electron density-based criteria responsible for the inhibitor potency. These features were calculated at the bond critical points determined in terms of the quantum theory of atoms in molecules (QTAIM) [38,39]. It was already shown that such an approach could explain macroscopic features of chemical reactions occurring in the active sites of enzymes [40,41,42,43,44]. We examined other known compounds with the same core and predicted their inhibition potency based on the correlation found for compounds with the known experimental IC50 values.

## 2. Models and Methods

**Figure 2:**
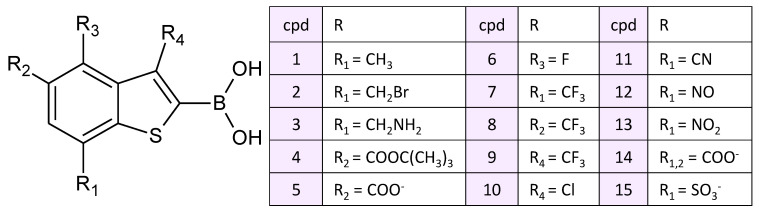
Boronic acid inhibitors considered in this study. Cpd1-cpd5 are experimentally studied in [26], cpd6-cpd10 are found in the PubChem [45] and cpd11-cpd15 are suggested in this study.

The model systems containing boronic acid inhibitors in the active site of NDM-1 were constructed using crystal structures PDB ID: 6IBV, 6Q2Y [35]. They corresponded to complexes of the NDM-1 with cpd2 and cpd3, respectively. The complexes with other experimentally studied boronic acid inhibitors, cpd1, cpd4 and cpd5 (Figure 2, compounds are numbered as in Ref. [26]) and other boronic acid-containing compounds with the benzo[b]thiophene core, cpd6-cpd15, were constructed by motifs of these X-ray structures. Substituted benzo[b]thiophene-containing boronic acids were found in the database of chemical compounds, PubChem (cpd6-cpd10, Figure 2) [45]. Additionally, we proposed ones with different electron-withdrawing groups (cpd11-cpd15, Figure 2).

The enzyme–substrate complex of NDM-1 and imipenem was obtained from the crystal structure PDB ID: 5YPK [12] with the hydrolyzed imipenem bound to NDM-1. The structure of the imipenem was restored to its non-hydrolyzed state.

In all complexes, hydrogen atoms were added using the Reduce program [46] to reproduce the protonation states of amino acids at neutral pH. The systems were solvated in rectangular water boxes so that the distance from the protein surface to the border of the cell exceeds 12 Å. These systems were neutralized by adding sodium or chloride ions. All atom force fields were utilized; CHARMM36 [47,48] for the protein, TIP3P [49] for water molecules and CGenFF [50] for the imipenem and inhibitors. Systems were equilibrated for 20 ns; the typical RMSD graph is shown in Figure S1. It was performed at T = 300 K and *p* = 1 atm with 1 fs integration time step. The substrate/inhibitor, catalytic OH^−^, zinc cations, side chains of amino acids forming coordination bonds with Zn^2+^ and a side-chain of Asp124 were fixed during this preliminary run. All MD simulations were performed in the NAMD program [51]. Last frames from the MD runs were utilized for the subsequent preparation of the QM/MM models. Water molecules located further than 6 Å from protein or 10 Å from the active site and sodium/chloride ions were removed from the model systems. Equilibrium geometry configurations were obtained at the QM(PBE0-D3/6-31G**)/MM(AMBER) [52,53,54,55] level of theory using the NWChem program [56]. An electronic embedding scheme was utilized with no cutoff on electrostatic interactions. The QM subsystem included the substrate or inhibitor, two Zn^2+^ cations and side chains of amino acid residues that form coordination bonds with them (His120, His122, His189, Cys208, His250), catalytic hydroxide anion and side chains of Asp124 and Asn220 that form hydrogen bonds with the substrate or inhibitor. Together, we obtained a set of 15 NDM-1 complexes with hydroxyboronate anion-containing compounds, EI. 

**Figure 3:**
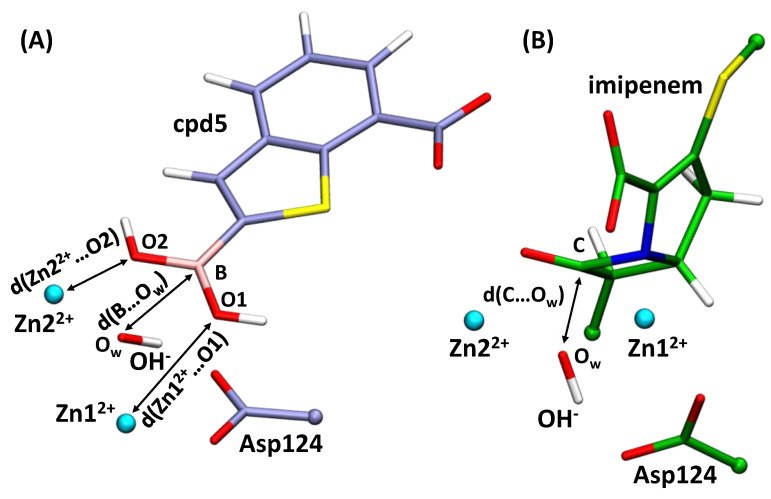
Complexes of NDM-1 and (**A**) cpd5, E·cpd5, or (**B**) imipenem, ES. Arrows indicate interatomic distances contributing to the reaction coordinate for the nucleophilic addition step. The reaction coordinates are ξ(E·cpd5) = d(Zn2^2+^…O2) + d(Zn1^2+^…O1) + r(B…O_w_) on the left panel and ξ(ES) = d(C…O_w_) on the right panel. For color code see Figure 1 caption. C_B_ atoms of the Asp124 are shown in balls. The imipenem substrate is truncated for clarity: the carbon atoms on the truncation borders are shown in balls.

Equilibrium geometry configurations of the NDM-1 with cpd5 (E·cpd5) and the ES complex after preliminary classical MD relaxation were utilized as initial structures for the molecular dynamics simulations with the QM/MM potentials. Solvation and neutralization of model systems, QM and MM partitioning and details of MD simulations are discussed above. The NAMD [51] program was utilized for MD steps performance and calculation of forces and energies of the MM subsystem. The TeraChem program [57] was used to calculate forces in the QM region, and a NAMD-TeraChem interface [58] was applied. The cutoff distance on the point charges of the MM subsystem contributing to the QM Hamiltonian was 12 Å. Gibbs energy profiles of the OH^−^ nucleophilic attack were calculated using the umbrella sampling approach. For the ES model, the reaction coordinate was the distance between the carbonyl carbon atom of the imipenem and the oxygen atom of OH^−^, ξ(ES) (Figure 3). Harmonic potentials ½·K·(ξ − ξ_0_)^2^ were centered at 1.3, 1.5, 1.7, 1.9, 2.1, 2.4, 2.6 and 2.8 Å with the force constant K = 40 kcal/(mol·Å^2^); additional runs were performed with potentials centered at 1.7, 1.8, 1.9 Å and K = 80 kcal/(mol·Å^2^); 1.7, 1.8, 1.9, 2.0, 2.1 Å and K = 120 kcal/(mol·Å^2^). A complex collective variable was suggested as a reaction coordinate of the nucleophilic addition reaction in the E·cpd5 complex, ξ(E·cpd5); it was set as a sum of two coordination bond distances, d(Zn2^2+^…O2) and d(Zn1^2+^…O1), and a distance of nucleophilic attack, d(B…O_w_) (Figure 3). Harmonic potentials were centered at the following ξ_0_ values: from 5.3 Å to 10.4 Å with 0.3 Å increment, from 10.5 Å to 10.8 Å with 0.1 Å increment, from 11.0 Å to 11.6 with 0.2 Å increment and 11.9 Å. The force constant was 40 kcal/(mol·Å^2^) in all simulations. The reaction coordinates were analyzed between 5 Å and 12 Å and divided into 200 bins in the case of E·cpd5 and between 1.3 Å and 3 Å with 50 bins for the complex NDM-1–imipenem complex. Each MD trajectory with additional harmonic potential added on the reaction coordinate was not less than 5 ps, and the first 1 ps was excluded from the data analysis. The weighted histogram analysis method (WHAM) was utilized to restore the Gibbs energy profile. The lengths of the QM/MM trajectories with harmonic potentials are considerably shorter than the conventional classical MD trajectories. However, we analyze the distributions that we obtain with each harmonic potential and check whether those changes with the further elongation of MD runs. Figures S2 and S3 depict distributions obtained with different harmonic potentials. MD trajectories of the comparable lengths were calculated in other enzymatic reaction mechanism studies; see, for example, Ref. [59]. Additional 10 ps runs were performed for the ES complex and E·cpd5 with the ξ_0_ = 10.1 Å and K = 40 kcal/(mol·Å^2^) harmonic potential.

Additional models were constructed for the cpd5 and imipenem (in the corresponding reactant states) solvated in the rectangular water box with the distance to the cell border being larger than 15 Å. The 1 ns preliminary classical MD simulation was performed before the 10 ps QM/MM MD run. Computational protocols were the same as discussed above.

Analysis of all MD trajectories and visualization of the equilibrium geometry configurations were performed in the VMD program [60].

We performed an analysis of electron density-based and geometry descriptors at the stationary points on potential energy surfaces and along QM/MM trajectories. In the latter case, we selected a set of 100 MD frames equally distributed along each considered MD trajectory. The electron densities were calculated, taking into account partial atomic charges from the MM subsystems that contributed to the one-electron part of the QM Hamiltonian. All electron density-based descriptors were calculated using the Multiwfn program [61]. We calculated electron density-based descriptors at bond-critical points (BCPs) corresponding to the coordination bonds between the zinc cations and hydroxyboronate anion-containing inhibitors (cpd1–cpd15) at minima on potential energy surfaces. These are electron density, *ρ*(r), and Laplacian of electron density, ∇^2^*ρ*(r). In addition, we calculate atomic contribution *S*(r, Ω*_i_*) [62,63,64] evaluated as

$$S\left(r,\Omega_{i}\right)=-\frac{1}{4\pi }\int_{\Omega_{i}}^{\;}\frac{\nabla^{2}\rho \left(r^{\prime }\right)}{\left|r-r^{\prime }\right|}dr^{\prime },$$

where Ω*_i_* is the atomic basin of the *i*-th atom; here, we consider oxygen atoms O1, O2 and O_w_ that are covalently bound to a boron atom (Figure 1). More details on the utilization of electron density-based descriptors for biologically relevant systems are described in Refs. [41,43]. In the case of QM/MM MD trajectories, we calculated atomic Fukui electrophilicity indices, f+, on a carbonyl carbon atom of the imipenem and a boron atom of cpd5. These are evaluated as differences between Hirshfeld charges [65] calculated for the model system with N electrons (as it is set in the calculations) and N + 1 electrons [66,67,68].

## 3. Results and Discussion

### 3.1. Imipenem and Boronic Acid Inhibitor Cpd5 in Water Solution and in the Active Site of NDM-1

The boronic acid inhibitors form covalent adducts with the catalytic OH^–^ ion as known from the crystal structures [26]. Here, we study the mechanism of this chemical reaction and compare it with the same nucleophilic addition step during the hydrolysis reaction of the imipenem substrate.

**Figure 4:**
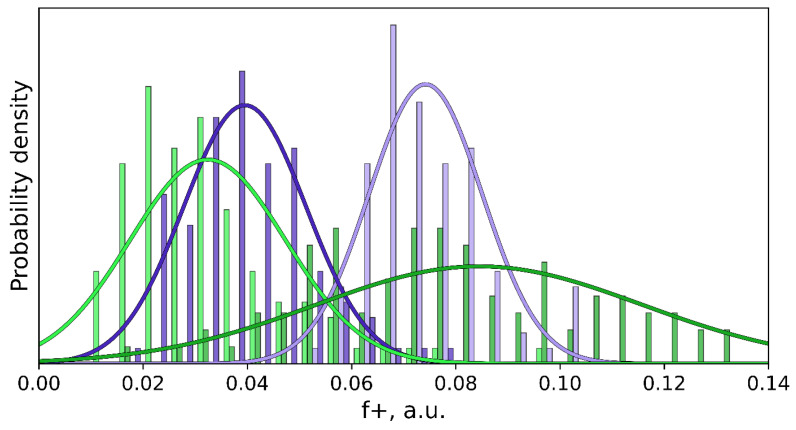
Distributions of Fukui electrophilicity indices, f+, on the carbonyl carbon atom of the imipenem (green) and boron atom of the cpd5 (violet) along the molecular dynamics trajectories with the combined quantim mechanics molecular mechanics potentials, QM/MM MD. Light colors correspond to the compounds in aqueous solutions, and dark colors are for the NDM-1 bound states. Corresponding normal distributions are shown in the same colors.

First, we analyze the behavior of cpd5 and imipenem in solution in terms of the electrophilicity of the boron and carbon atoms that form covalent bonds with the OH^−^ during the reaction. We use the Fukui function for the electrophilic attack, f+, calculated for the C (in the imipenem) and B (in the cpd5) atoms along the MD trajectories (Figure 4). The f+ distribution is considerably shifted to the larger values in the case of B, indicating that it is a stronger nucleophile in the solution. The f+ value for the carbonyl carbon atom in the imipenem is 0.032 ± 0.015 a.u. and 0.074 ± 0.011 a.u. for B of the cpd5.

**Figure 5:**
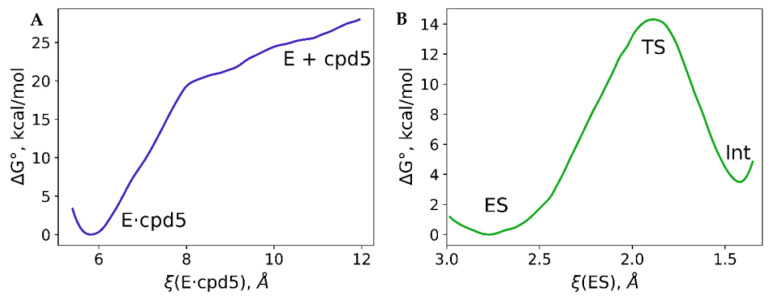
Gibbs energy profiles of the nucleophilic attack step in the active site of NDM-1 calculated (**A**) for the boronic acid-based inhibitor cpd5 and (**B**) for the imipenem substrate. The description of the reaction coordinate is shown in Figure 3 and in the Methods section.

Next, we study the behavior of these two compounds in the active site of NDM-1. We constructed the ES complex with an imipenem molecule. During the QM/MM MD simulation, the distance of the nucleophilic attack varied in the range of 2.89 ± 0.15 Å. The mean value of the f+(C) increases more than twice compared with water solution being 0.084 a.u (Figure 4). This distribution is considerably broader, with the standard deviation being 0.031 a.u. that is twice larger than in solution. It means that not all ES complexes (frames along the MD trajectory) correspond to the reactive species. The fraction with the larger f+ values demonstrates the substrate activation in the active site of the enzyme discussed in Refs. [59,69]. The Gibbs energy profile for the nucleophilic attack step was calculated using the umbrella sampling technique (Figure 5). The minimum corresponding to the ES complex is located at the reaction coordinate value of 2.79 Å. It is slightly shorter than the mean C…O_w_ distance along the MD trajectory of the ES complex. During the unconstrained MD simulation of the ES complex, we observe both reactive and nonreactive species, whereas only reactive species contribute to the chemical reaction [43]. The C…O_w_ distance at the transition state is 1.92 Å that is typical for this type of reaction occurring in the zinc-dependent proteins. To compare, it is 1.86 Å in the case of nitrocefin hydrolysis by L1 metallo-β-lactamase [70] and 1.80 Å during the oligopeptide hydrolysis by MMP-2 matrix metalloproteinase [71]. The energy barrier at this step is about 14 kcal/mol, and the first intermediate, Int, is ~4 kcal/mol higher in energy than the ES complex.

The Gibbs energy profile of the covalent bond formation between the OH^−^ and the cpd5 was studied within the same QM/MM protocol. First, we performed the QM/MM MD run of the product state (E·cpd5) with the covalent bond between the cpd5 and OH^−^. We gradually increased the reaction coordinate and performed sequential umbrella sampling runs. The first attempt was a simple reaction coordinate, d(B…O_w_), similarly to the reaction with the imipenem. However, this resulted in the artificial behavior of the model system. Therefore, we proposed a more complex reaction coordinate that accounts not only for the distance of nucleophilic attack but also for two coordination bonds between oxygen atoms, O1 and O2, of the cpd5 and zinc cations (Figure 3). The reaction coordinate equals 5.8 Å at the minimum, corresponding to the covalent complex E·cpd5 with two coordination bonds, Zn1^2+^…O1 and Zn2^2+^…O2, and a covalent B–O_w_ bond being formed. The dissociation energy profile has two regions. A smaller reaction coordinate values, the rapid increase of energy results in the cleavage of the covalent bond B–O_w_ and elongation of coordination bonds. Starting from the ξ = 8 Å, the energy increases much slower; the covalent bond is already cleaved, and the dissociation process is mostly related to the complete cleavage of coordination bonds. One should keep in mind that even at the ξ = 12 Å, the NDM-1 and cpd5 complex is not fully dissociated. More precisely, the coordination bonds between the cpd5 and zinc cations are cleaved, but components of this complex are not fully solvated. We expect that at larger ξ values, the Gibbs energy will decrease.

**Figure 6:**
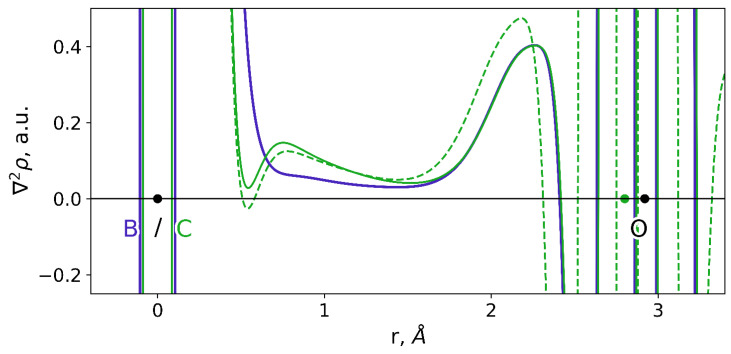
Laplacian of electron density, ∇^2^*ρ*(r), along the C/B…O_w_ direction of the nucleophilic attack. Solid curves correspond to the NDM-1–imipenem (green) and NDM-1–cpd5 (violet) with the same distance of nucleophilic attack. The dashed green curve corresponds to another snapshot along the NDM-1–imipenem complex QM/MM MD trajectory. Boron and carbon atoms are always located at the *r* = 0 Å value; an oxygen atom is shown in black if it occupies the same position for complexes with both imipenem and cpd5 and colored green for another ES snapshot with the ∇^2^*ρ*(r) shown in green dashed line.

We failed to obtain a stable prereaction complex of the NDM-1 and the cpd5. However, we analyzed the dynamic behavior of the system in the geometry configuration that is similar to the ES complex. We performed a constrained MD run with the ξ_0_ = 10.1 Å and the force constant imposed on the reaction coordinate K = 40 kcal/(mol·Å^2^). The B…O_w_ distance varied in the range of 2.97 ± 0.13 Å. We expected that the f+ on the boron atom is higher than in the solution. However, it turned out that it was considerably smaller and similar to that of a carbon atom of imipenem in the solution being 0.040 ± 0.012 a.u. (Figure 4). To analyze this unexpected phenomenon, we calculated the Laplacian of electron density, ∇^2^*ρ*(r), along the C…O_w_ and B…O_w_ at different frames. Figure 6 demonstrates the alignment of ∇^2^*ρ*(r) for complexes of the NDM-1 with the cpd5 or the imipenem with the same distances of the nucleophilic attack. The ∇^2^*ρ*(r) curves are similar around the oxygen atom of the OH^−^ and are different in the B/C region. In the case of a carbon atom, we observe a minimum at ~0.5 Å and a maximum at ~0.7 Å from a C nucleus. In the case of a boron atom, we observe a flat region between two atoms with the small deconcentration of electron density (∇^2^*ρ*(r) > 0).

We analyzed the ∇^2^*ρ*(r) curves at different MD frames. For different NDM-1 with cpd5 complexes, we observe similar behavior of the Laplacian of electron density in the interatomic region: a flat area with the electron density deconcentration, with ∇^2^*ρ*(r) being around 0.05 a.u. Such regions are attributed to the presence of the substrate activation effect that was previously demonstrated on the 2D maps of ∇^2^*ρ*(r) [42,43,69,72]. From this viewpoint, the boron atom is activated in all presented MD frames. However, its Fukui electrophilicity index is lower when bound to NDM-1 compared with the aqueous solution. Similar abnormal behavior of a boron atom is discussed in Ref. [73] and it is attributed to the strong *p*-π electronic interactions; that is, the empty *p*-orbital of a boron center is partly filled by the π-electron of the neighboring atom. In our case, the partner of these interactions is a nucleophilic OH^−^ that partly “donates” its electron lone pair to the empty boron *p*-orbital. It seems that these interactions, together with the long-range electrostatic interactions between the coordination bond partners, may be the reason for the failure to locate a minimum corresponding to the prereaction complex.

Laplacian of electron density curves for the ES complex at different MD frames are diverse. Depending on the particular frame, a minimum that is 0.5 Å apart from a carbon atom has either a positive (solid green line on Figure 6) or negative (green dashed line on Figure 6) ∇^2^*ρ*(r) value. It means that a substrate is activated only at certain frames. It is in line with the presence of equilibrium between the reactive and nonreactive species [43].

### 3.2. Interatomic Interactions Responsible for the Inhibition Potency

**Figure 7:**
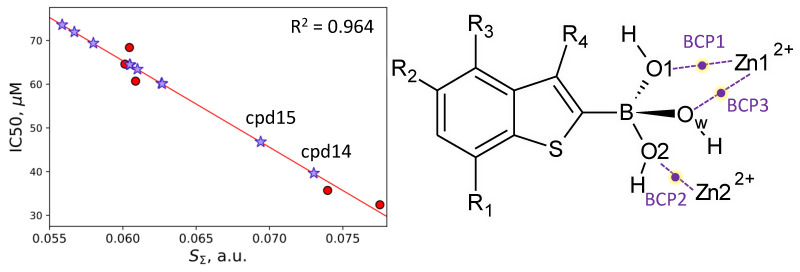
The IC50 = f(S_Σ_) dependency; red dots correspond to cpd1–cpd5 inhibitors; cpd6–cpd15 are marked with lavender stars. Prospective compounds, cpd14 and cpd15, are named. Coordination bonds between the hydroxyboronate anion and zinc cations are shown with violet dashed lines, and corresponding bond critical points (BCPs) are highlighted yellow.

Another important issue is the prediction of the inhibitor potency of boronic acid compounds. We calculated equilibrium geometry configurations for a set of covalent complexes of NDM-1 and hydroxyboronate anion-containing compounds with available experimental IC50 values [26]. All complexes demonstrate similar geometry features. The length of the newly formed covalent bond between a boron-based inhibitor and a former nucleophilic hydroxide anion, B–O_w_, is 1.45–1.49 Å; covalent B–O1 and B–O2 bonds lengths are distributed similarly. Coordination bonds, Zn1^2+^…O_w_, Zn1^2+^…O1 and Zn2^2+^…O2 are 1.94–1.96 Å, 2.32–2.51 Å and 1.95–2.03 Å, respectively (Table 1, Figure 3). Here, the QTAIM [38,39] is utilized to identify and characterize interatomic interactions. It was already demonstrated that even in large systems, such as enzymes, one could identify key interatomic interactions that are responsible for the observed macroscopic property [41]. The first step in the search of key interatomic interactions is interatomic distances check. Geometry parameters demonstrate a worse correlation. However, if these exist, this can be the hint for electron density-based descriptor search. We found that electron density-based descriptors at the bond critical points (BCPs) of the coordination bonds between the hydroxyboronate anion and zinc cations correlate with the IC50 values (Table 1, Figure 7). These are Zn1^2+^…O1, Zn2^2+^…O2 and Zn1^2+^…O_w_ coordination bonds with the corresponding BCP1, BCP2 and BCP3. We obtained QSPR equations between the IC50 values and coordination bond length, electron density at BCP, Laplacian of electron density at BCP and atomic contribution of an oxygen atom forming coordination bond to a corresponding BCP (Table 1). Sums of corresponding values at BCP1, BCP2 and BCP3 were also examined. The best correlations were obtained for the IC50 dependency on the atomic contributions at BCP and sums of descriptors calculated at three BCPs. These QSPR equations were further utilized for the prediction of the IC50 values of new compounds.

NDM-1 complexes were modeled with compounds cpd6–cpd10 presented in the PubChem [45] and having the same benzo[b]thiophene core. However, their predicted IC50 values were found to be higher than 60 μM. Therefore, we returned to the experimentally studied set. The cpd5 has the lowest value of IC50 among those studied experimentally, so it was chosen as a lead compound to design new inhibitors. Namely, we proposed that electron-withdrawing groups are responsible for the IC50 value. We suggested and examined another set of compounds (cpd11–cpd15), replacing the carboxyl group in cpd5 with other electron-withdrawing groups or adding them at different positions. All of them demonstrated IC50 values in the same range. Among them, the most promising are cpd14 and cpd15 (Figure 2), which have predicted IC50 values 40 μM and 47 μM, respectively (Figure 7). It seems that a further decrease of the IC50 value can be achieved if changing the core of boronic acid compounds.

## 4. Conclusions

Application of molecular dynamics simulations with the QM/MM potentials complemented with on-the-fly calculations of electron density-based descriptors allowed us to study and compare nucleophilic attacks of the catalytic species in the NDM-1 active site. We compared the reaction mechanism with a substrate imipenem and a boronic acid inhibitor. The nucleophilic attack occurs with the energy barrier of 14 kcal/mol in the case of the imipenem and simultaneously upon binding of an inhibitor. Activation of the carbonyl carbon atom of the imipenem is observed in the enzyme–substrate. The boronic acid compound is already activated, even being surrounded by water molecules. These properties are quantified by the atomic Fukui electrophilicity indices. Binding to the active site of NDM-1 comprises both formations of the prereaction interactions between the boron atom of an inhibitor and a catalytic OH^−^ and coordination bonds between OH groups of an inhibitor and zinc cations. The latter interactions are predominantly of an electrostatic nature and are long-range. Upon the formation of the prereaction complex between NDM-1 and inhibitor, the lone electron pair of the nucleophile interacts with the vacant *p*-orbital of boron that facilitates the chemical reaction. This explains the barrierless process of covalent bond formation between an OH^−^ and a boronic acid if a latter comes to the active site of NDM-1.

We analyzed a set of boronic acid compounds with the benzo[b]thiophene core complexed with the NDM-1 and proposed QSPR equations to predict IC50 values from the calculated descriptors of electron density. Among them, the prospective ones are quantities obtained as sums of electron densities, Laplacian of electron densities or atomic contributions calculated at BCPs of three coordination bonds between oxygen atoms of hydroxyboronate anion and zinc cations. We examined a set of compounds with the same benzo[b]thiophene from the PubChem database and proposed others ourselves. The predicted IC50 values were found to be larger than 30 μM, similar to those studied experimentally. We suppose that a further decrease of this value can be achieved if changing the core of boronic acid compounds.

## Figures and Tables

**Table 1:** Characteristics of linear dependences between descriptors at BCPs of coordination bonds and experimental IC50 values. The slope units are reciprocal descriptor units multiplied by μM. The Σ subscript in the last three rows corresponds to the sum of corresponding descriptors at BCP1, BCP2 and BCP3 that are located on the bond paths between Zn1^2+^ and O1, Zn2^2+^ and O2, Zn1^2+^ and O_w_, respectively. RSS is the residual sum of squares.

Descriptor	Descriptor Range	Slope	Intercept, μM	R^2^	Error, μM	RSS, μM^−2^
d(Zn1^2+^…O1), Å	2.32–2.51	184 ± 41	−397 ± 100	0.827	195–203	150
*ρ*(r_BCP1_), a.u.	0.025–0.038	−2667 ± 618	131 ± 19	0.815	34–42	160
∇^2^*ρ*(r_BCP1_), a.u.	0.11–0.17	−644 ± 159	137 ± 21	0.793	39–48	179
S(r_BCP1,_ O1), a.u.	0.0018–0.0081	−5489 ± 1238	74 ± 6	0.823	8–16	153
d(Zn2^2+^…O2), Å	1.95–2.03	415 ± 51	−778 ± 102	0.942	202–206	50
*ρ*(r_BCP2_), a.u.	0.072–0.094	−1643 ± 259	183 ± 21	0.908	39–45	80
∇^2^*ρ*(r_BCP2_), a.u.	0.28–0.34	−602 ± 102	234 ± 31	0.894	60–66	92
S(r_BCP2,_ O2), a.u.	0.026–0.036	−3413 ± 432	152 ± 13	0.939	24–28	53
d(Zn1^2+^…O_w_), Å	1.94–1.96	1094 ± 640	−2088 ± 1251	0.325	2490–2507	583
*ρ*(r_BCP3_), a.u.	0.088–0.094	−3348 ± 3930	354 ± 354	–	700–723	927
∇^2^*ρ*(r_BCP3_), a.u.	0.33–0.36	−1207 ± 569	458 ± 191	0.467	378–393	461
S(r_BCP3,_ O_w_), a.u.	0.033–0.036	−8964 ± 5351	352 ± 179	0.311	353–370	595
*ρ*_Σ_, a.u.	0.19–0.22	−1017 ± 114	255 ± 23	0.952	44–48	42
∇^2^*ρ*_Σ_, a.u.	0.72–0.84	−292 ± 26	277 ± 20	0.968	39–43	28
S_Σ_, a.u.	0.060–0.078	−1979 ± 192	184 ± 13	0.964	24–28	31

## Data Availability

Equilibrium geometry configurations of EI complexes are available at the ZENODO at https://doi.org/10.5281/zenodo.4606675 (accessed on 1 April 2021).

## Supplementary Materials

The following are available online, Figure S1: RMSD along the classical MD trajectory, Figures S2 and S3: reaction coordinate distributions along the QM/MM MD simulations with the harmonic potentials.
