# Supplementary material for: Boronic Acids as Prospective Inhibitors of Metallo-β-Lactamases: Efficient Chemical Reaction in the Enzymatic Active Site Revealed by Molecular Modeling
Source: Molecules. 2021 Apr 2;26(7):2026. doi: 10.3390/molecules26072026 (PMC8038151; doi:10.3390/molecules26072026)
Supplement: Supplementary file 1 [file molecules-26-02026-s001.pdf]

Supplementary materials for

Boronic acids as Prospective Inhibitors of Metallo-β-Lactamases: Efficient Chemical Reaction in the Enzymatic Active Site Revealed by Molecular Modeling

Alexandra V. Krivitskaya [1] and Maria G. Khrenova [1,2,*]

[1]Bach Institute of Biochemistry, Federal Research Centre "Fundamentals of Biotechnology" of the Russian Academy of Sciences, Moscow, 119071, Russia; al_krivickaya@mail.ru (A.V.K.)

[2]Department of Chemistry, Lomonosov Moscow State University, Moscow, 119991, Russia; mkhrenova@lcc.chem.msu.ru (M.G.K.)

* Correspondence: mkhrenova@lcc.chem.msu.ru

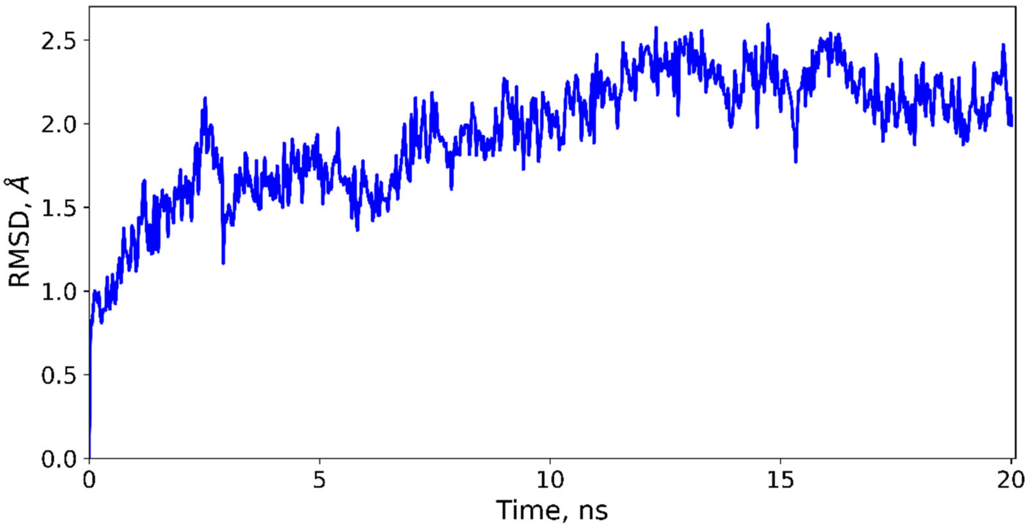

Figure S1. The RMSD calculated over protein backbone along the 20 ns MD trajectory of the ES complex.

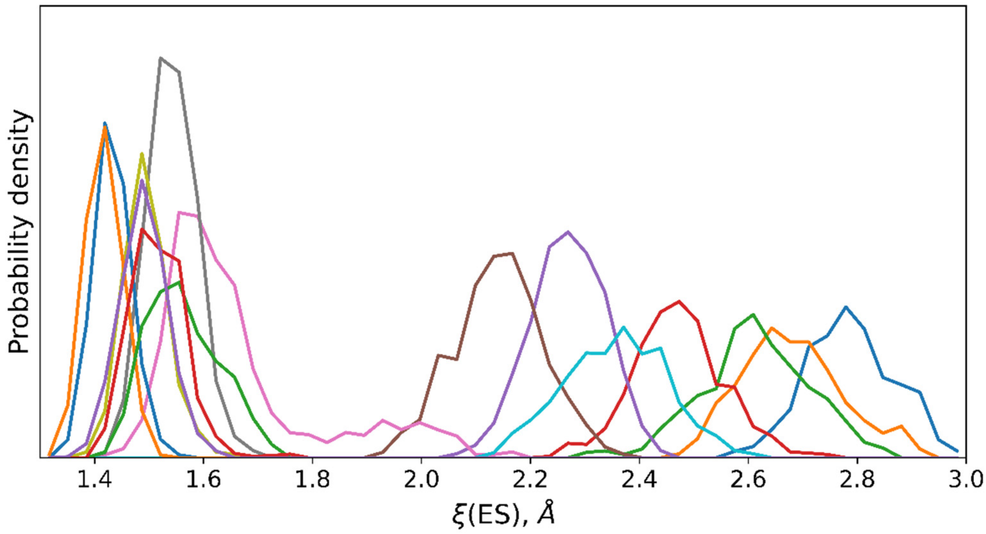

Figure S2. Distributions of the reaction coordinate, ξ(ES), calculated along MD trajectories with different harmonic potentials.

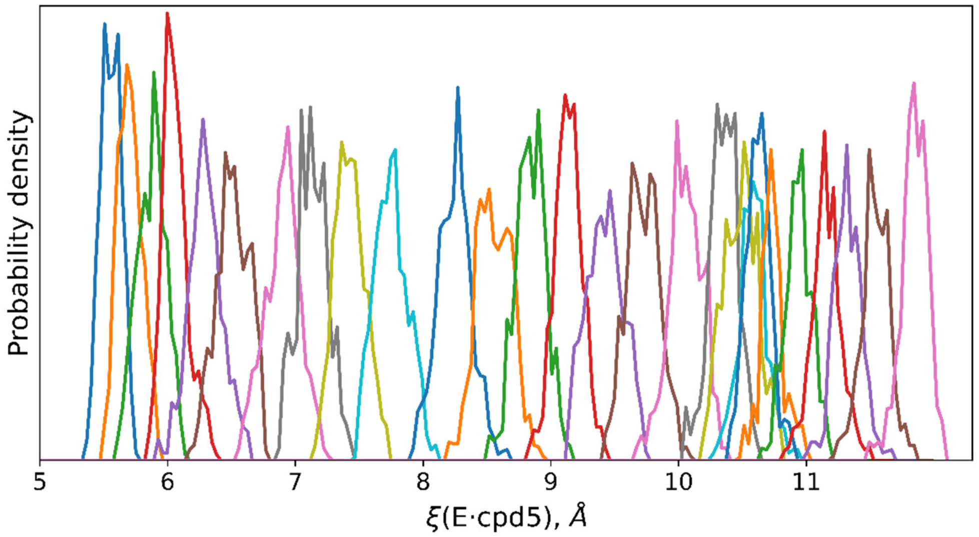

Figure S3. Distributions of the reaction coordinates, ξ(E·cpd5), calculated along MD trajectories with different harmonic potentials.
